# “The mosquitoes that destroy your face”. Social impact of Cutaneous Leishmaniasis in South-eastern Morocco, A qualitative study

**DOI:** 10.1371/journal.pone.0189906

**Published:** 2017-12-20

**Authors:** Issam Bennis, Loubna Belaid, Vincent De Brouwere, Hind Filali, Hamid Sahibi, Marleen Boelaert

**Affiliations:** 1 National School of Public Health—Ministry of Health, Rabat, Morocco; 2 Department of Public Health—Institute of Tropical Medicine, Antwerp, Belgium; 3 Department of Biomedical Sciences—Faculty of Pharmaceutical, Biomedical and Veterinary Sciences- University of Antwerp, Antwerp, Belgium; 4 CRCHUM, École de Santé Publique de l’Université de Montréal, Montréal, Canada; 5 Agronomy and Veterinary Institute Hassan II, Rabat, Morocco; McGill University Faculty of Education, CANADA

## Abstract

**Objective:**

To document the psychosocial burden of Cutaneous Leishmaniasis (CL) in rural communities in Southeastern Morocco.

**Method:**

Between March and April 2015, we conducted qualitative research in communities exposed to *Leishmania major* or *L*. *tropica* in Errachidia and Tinghir provinces. Twenty-eight focus groups discussions (FGDs) were realized, with a stratification by gender and tradition of medicine (users of folk versus professional medicine). Data were analyzed using content analysis.

**Results:**

This rural population most exposed to CL in Morocco lacks access to health care in general and clearly points out there are other major public health issues that need to be resolved. Nonetheless, respondents consider the impact of CL lesions and scars as important and similar to that of burn scar tissue. Young women with CL scars in the face are stigmatized and will often be rejected for marriage in these communities. People usually try a long list of folk remedies on the active lesions, but none was felt adequate. There was a clear demand for better treatment as well as for treatment of the scars.

**Conclusions:**

The psycho-social impact of CL due to *L*.*major* and *L*.*tropica* is substantial, especially for young single women with facial scars. These generate social and self-stigma and diminish their marriage prospects. CL is well known, but not considered as a major health priority by these poor rural communities in South-eastern Morocco where gender discrimination is still an issue and access to basic health care is as neglected as CL. Early CL diagnosis and new treatment options with better skin outcomes are urgently needed.

## Introduction

Leishmaniasis is a vector-borne disease caused by protozoan species of the genus *Leishmania* and transmitted by different sand fly species of the *Phlebotomus* and *Lutzomyia* genus. The disease manifests as one of three main clinical presentations: visceral, cutaneous or mucocutaneous [1, 2]. Cutaneous Leishmaniasis (CL) is quite common in Morocco and is caused by the anthroponotically transmitted *L*.*tropica* or the zoonotic *L*.*major* species [3, 4]. In some areas both causal species coexist [5, 6]. CL has led to epidemic outbreaks in Morocco in the past, the most recent one occurring between 2004 and 2013 in the Southeast of the country with more than 10,000 recorded cases [7, 8].

**Fig 1:**
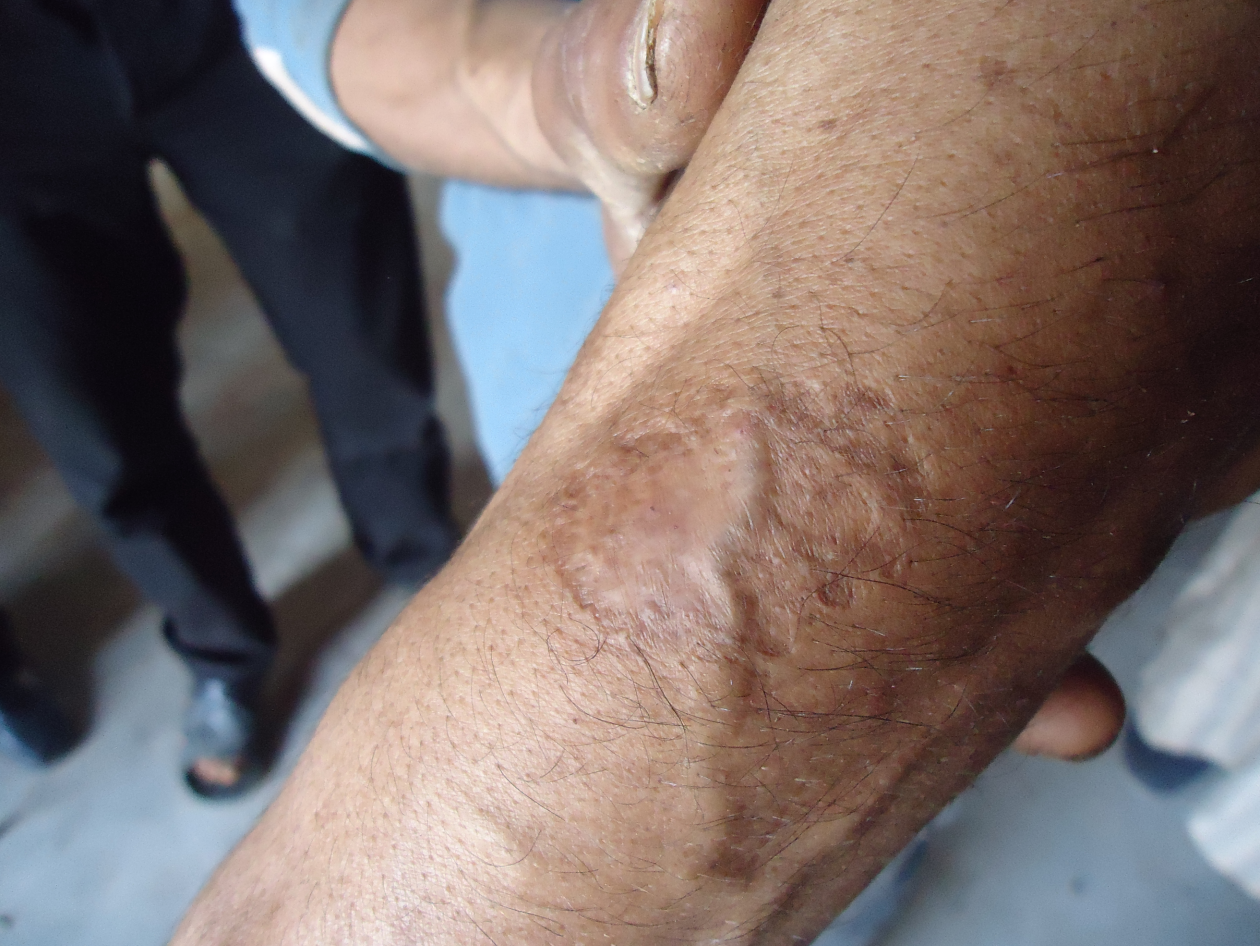
CL scar on the forearm of a man living in a *L*.*major* endemic area in South-eastern Morocco (Picture taken by IB).

**Fig 2:**
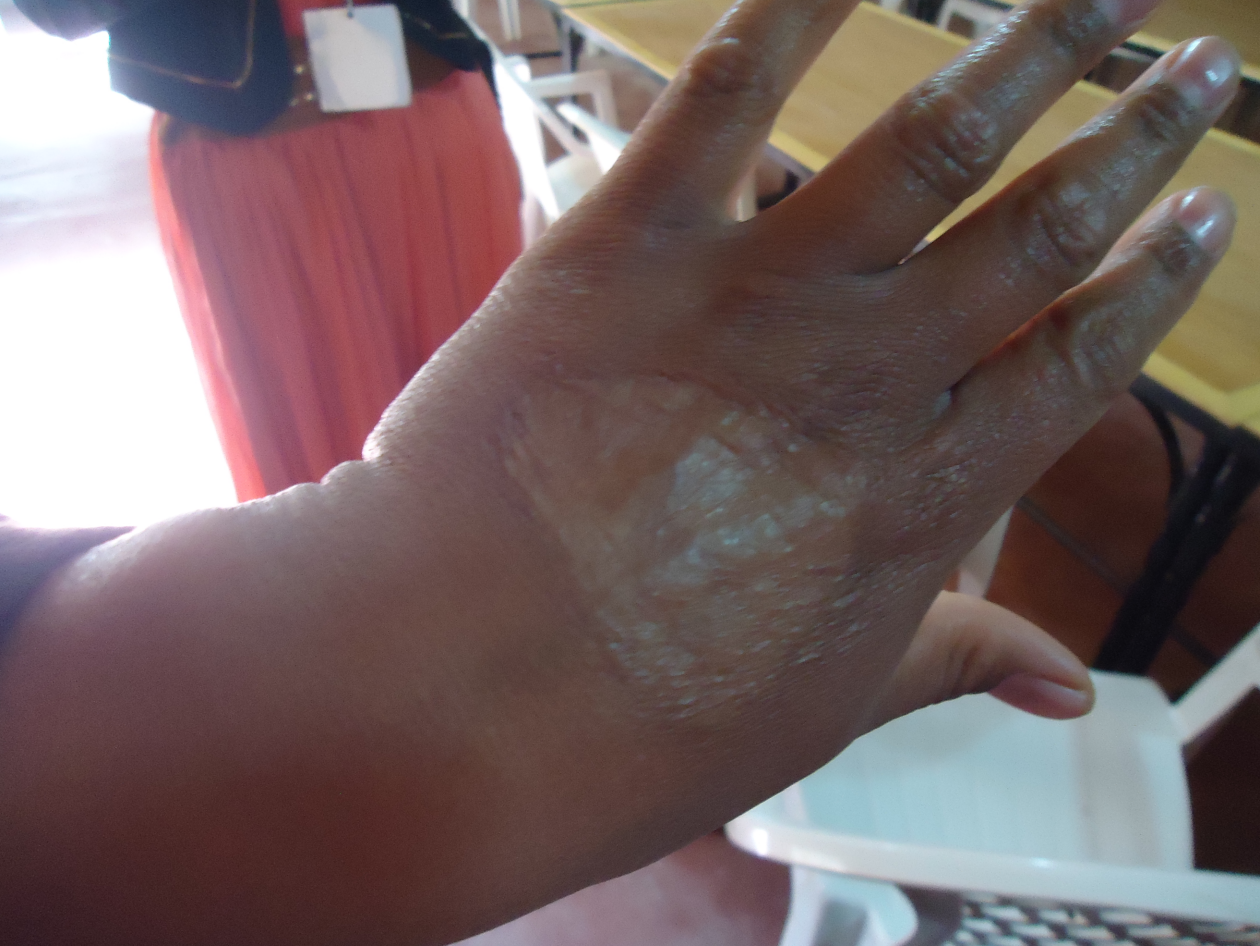
CL scar on the hand of a woman living in a *L*.*major* endemic area in South-eastern Morocco (Picture taken by IB).

Most CL lesions develop on the uncovered parts of the body as those are most exposed to the sand fly bites [9]. In Moroccan patients CL lesions are of the “localized” type and start as one or multiple slowly progressing nodules that subsequently ulcerate. The healing of this skin ulcer usually leads to substantial scar tissue, which can be quite disfiguring (Figs 1 and 2). CL tends to be considered as a minor illness, when compared to the visceral and mucocutaneous forms of leishmaniasis that have fatal or severely mutilating outcomes. However, little research has actually been done about how the affected communities perceive CL, what their explanatory model is for it, and how they deal with this illness. A number of Knowledge, Attitude and Practices (KAP) surveys in different countries and age groups showed a general lack of knowledge regarding CL symptoms, transmission mode, and reservoir [10–18]. We observed in our previous work in adolescent youth living in the region affected by the CL epidemic that in this age group the psychological impact is real and the demand for prevention and care, especially for the repair of scar tissue was clearly formulated [19]. We wanted to expand this work to the general population, as a contribution to the formulation of sound control CL policy.

The aim of this study is to describe the social burden generated by CL in Morocco, by exploring the community perspective on this illness and social consequences related to CL in the adult population living in rural endemic areas in South-eastern Morocco.

## Methods

We followed the Consolidated Criteria for Reporting Qualitative research (COREQ) to report our findings. A checklist is provided as supporting information (S1 File).

### Research team

Our research team was multidisciplinary, composed of three public health experts with a medical background, two psycho-social scientists and one veterinary scientist. The principal investigator (IB), who is a male Arabic-speaking researcher with a medical background and special training in qualitative research methods, conducted the field work. He was not involved in clinical care in the area and was not known to the study participants. He was accompanied by male health worker or a female community member known to the respondents.

### Study design

#### Study population

**Fig 3:**
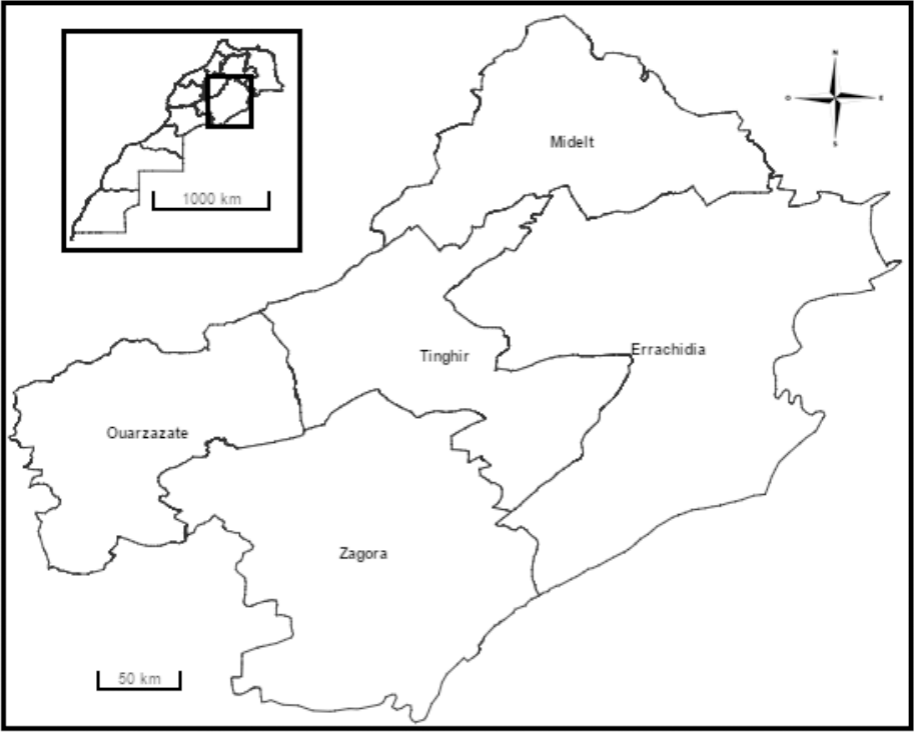
Map showing Tinghir and Errachidia provinces located in Deraa-Tafilalet Region, Morocco. (Source www.hcp.ma).

Our primary aim was to describe the community perspective on CL following a qualitative explanatory case study approach [20]. We conducted Focus Group Discussions (FGDs) between March and April 2015 in two provinces in South-eastern Morocco: Errachidia and Tinghir (Fig 3), the first endemic for *L*.*major* and the second predominantly for *L*.*tropica* [7, 21]. Table 1 shows the recently reported case load of CL by species in these provinces. According to the High Commission for Planning in Morocco, poverty rates in both provinces are high (18–24%) and unemployment rates (9–16%) are higher than in other regions of Morocco.

**Table 1:** Number of cases of CL due to *L*.*major* and *L*.*tropica* in south eastern Morocco as notified to the Ministry of Health (2013–2016).

	*L*.*major* cases					*L*.*tropica* cases				
	2013	2014	2015	2016	Total	2013	2014	2015	2016	Total
**Errachidia Province**	11	12	231	896	1150	0	0	0	0	0
**Tinghir Province**	155	90	116	342	703	337	244	110	504	1195
**Ouarzazate Province**	89	170	358	353	970	0	85	61	102	248
**Morocco, total**	537	460	954	1889	3840	2054	2094	1859	3062	9069

In each province, we purposefully chose the two districts with the highest number of CL cases notified between 2009 and 2013. Within each district, we used snowballing as purposive sampling strategy to select study participants from the CL endemic areas aiming for maximum diversity in age, gender, and socioeconomic background. We pre-planned a stratification of the FGDs on two dimensions: gender and cultural preference for tradition of medicine (folk or allopathic medicine). The purpose of this stratification was to allow a free and uninhibited discourse within groups. The users of the allopathic sector were invited by approaching people who accompanied their relatives to the health centers in villages. To reach the people who prefer folk healers, we used a snowballing approach starting from key informants in the villages who put us in touch with traditional healers and their networks.

In total, we conducted sixteen FGDs: in each of the four districts there were four FGDs: one with male and one with female clients of the formal health sector (henceforward indicated as ‘allopathic’), and one with male and one with female users of folk medicine (‘folk’). Based on our findings in Errachidia province however, we decided to add twelve more FGDs to include the perspectives of specific communities that were living in very remote and difficult to reach areas. A total of 251 participants attended the 28 FGDs.

FGDs included between seven and fourteen participants in each session. Four men and ten women in Tinghir and seven men and nineteen women in Errachidia, all users of the allopathic sector, refused our initial invitation. Reasons invoked were a lack of time, impossibility to leave their children in the care of someone else, or failure to obtain approval from their spouses. No participant received any incentive.

We organized the FGD sessions based on what our respondents proposed as suitable time and location. The discussions took place in quiet rooms that provided sufficient privacy, such as coffee houses, mosques, schools, private houses or local NGO offices.

#### Data collection

During the FGD we used a topic guide (S2 File) inspired by Brown *et al*., [22] who analyzed the determinants of quality of life in patients with scar tissue. Topics included knowledge of CL, physical and psychological consequences, behavior, social impact, treatment outcome and knowledge of preventive measures. After completion of the first two FGD, we adapted the topic guide slightly, as we wanted to better understand the perception of CL scars and the attitude/behavior towards people with CL scars better. Thus, we added two questions: ‘How can you make the scars go away?’ and ‘How do people in general behave with those affected with this disease?

The two main languages used for these discussions were the Amazigh (local language) and Moroccan Arabic. Each FGD was recorded with audio and video recording except for three FGDs for which we only used audio recording at the request of the participants. At the end of each FGD, the researcher took notes after discussion with the observer. An average of 39 minutes (range 28–64) duration was observed for the initial 16 FGDs and an average of 31 minutes for all FGDs (Table 2).

**Table 2:** Characteristics of focus groups and participants.

	Tinghir Province	Errachidia Province	Additional FGD *
**Health facility (Allopathic) clients**			
Number of FGD	4	4	6
FGD mean duration in minutes (min-max)	41 (31–60)	32 (28–35)	18 (15–23)
Total number of female participants [age range in years]	16 [19–46]	22 [19–58]	23 [19–68]
Total number of male participants [age range in years]	16 [24–57]	22 [21–60]	22 [20–67]
**Traditional medicine (Folk) clients**			
Number of FGD	4	4	6
FGD mean duration in minutes (min-max)	42 (37–46)	40 (29–64)	23 (14–32)
Total number of female participants [age range in years]	24 [21–82]	19 [19–58]	23 [19–68]
Total number of male participants [age range in years]	16 [24–57]	22 [21–60]	22 [20–67]

FGD: Focus group discussion.

(*) done in Errachidia province to reach saturation.

### Data analysis

We followed the process of directed content analysis to analyze the text data [23]. FGDs were transcribed and translated into French. Two persons (IB, HF) independently coded data, one using NVivo 10 software and the other one coded manually. We adopted a single coding framework, including themes and subthemes, see Table 3. The codes were informed by our previous work targeting the adolescents [19] and the conceptual model by Brown et al., 2008 [22]. We used the main themes as a structure for reporting our findings.

**Table 3:** Themes and subthemes in the coding framework.

**CL Knowledge**	**Gender discrimination**
CL name	Marriage opportunities
CL causes	Stigma
CL symptoms	Coping strategies
CL transmission ways	Prevention strategies
**CL Perception**	**Health seeking patens for CL**
CL severity	Treatment accessibility
Skin outcome	CL traditional remedies
Psychological effects	CL conventional therapies
	CL scar treatment

### Ethical considerations

The Institutional Review Board of the Institute of Tropical Medicine in Antwerp Belgium (956/July.2014) and the Ethical Committee of Biomedical Research of the Faculty of Medicine and Pharmacy in Rabat Morocco (1139/04_Nov.2014) have approved the study. We obtained the authorisation to conduct the study from the national and local Ministry of Health authorities.

Participation in the FGD was entirely voluntary. Invited participants were given an information sheet (S3 File) to read, and asked to present themselves at the appointment for the FGD at a specific day and time if they agreed to participate. At the specific setting, their oral consents were recorded on audiotape. The participant’s identities were anonymised in the data. Feedback of our findings will be given to the communities after the results of this work will be published.

## Results

Without having put ‘young age’ as an exclusion criterion, all respondents in our FGD were more than 19 years old, and this was the case in both male and female groups (see Table 2). The FGD participants did not allow the young and adolescents to sit with them and to participate in this discussion. We present the results within four themes: CL knowledge; CL perception; gender discrimination; health seeking patterns for CL.

### CL knowledge

The people in this region know CL as the “*disease of the scars*” and describe the natural evolution rather accurately, as a spot or several bumps on the skin you develop after a mosquito bite, sometimes blistering or ulcerating but not healing for four to six months and eventually leading to a permanent bluish/black mark or scar. In this rural society, almost everybody has some scar tissue, but the local names for CL as the “*mosquito sore*” or the “*mosquito scar*” clearly distinguish them and link the skin lesion to an insect vector. All FGDs said that the sore was caused by the bite of a mosquito (*chniwla*), and the^,^ distinction between mosquito and sand fly was not made. However, the causal chain between an insect bite leading to CL and later scar tissue was very clear for everybody. One person summarized this as “*the mosquitoes that destroy your face*”. Other names were used as well, usually referring to the locality where the problem was first observed, such as “*sore of Touroug*”.

Respondents also spontaneously used the terms ‘*leishmania*’ or ‘*leishmaniasis*’. In Errachidia province, the term seemed familiar to many in all the allopathic FGD groups and in some of the folk medicine ones. Contrastingly, in Tinghir, the term ‘leishmaniasis’ was only used in two allopathic FGD and not in the others. People in Tinghir seemed less familiar with CL in general and did not think it was common in their area, whereas in Errachidia many respondents had experienced CL themselves or had relatives and friends affected by it.

FGDs respondents stated that CL is not contagious by direct contact, nor transmitted by water. Stagnant water, manure, dead rats and pollution, in general, were repeatedly evoked as risk factors, as they increase mosquito density. People acknowledge that the risk is seasonal, and higher in summer. In summer, people tend to sleep outside which exposes them to more mosquito bites. Insect nuisance was said to be substantial in the villages, and a quiet night of sleep hard to get by because of this. Women were considered most exposed to mosquito bites as they engage in farming activities taking care of cattle and dealing with manure and waste. Little children and tourists were thought to be more vulnerable as well, and a few respondents attributed this to their lack of immunity, sweeter blood or softer skin.

### CL perception

Several women in the Errachidia area compared the severity of CL to that of acne and stated that CL is more severe. *“Leishmania is more unpleasant than acne*. *Acne is not pitting*, *but leishmania digs the skin”* (FGD15 Women-Allopathic). In some cases, the lesions can be truly incapacitating depending on the location, such as eyes, foot soles or hands. Active lesions on the hands impair the daily work of women. *“If the disease is not on the hands you use to prepare the bread and meals*, *it's not a problem*.*”* (FGD10 Women-Folk).

All FGDs agreed that the scar tissue of CL can be a real problem, mostly for women. *“The most serious is the scar in the middle of the face*. *If it is on the side of the face it can be hidden by the veil”* (FGD26 Women-Allopathic). A CL scar was considered similar to a burn scar: “*The scar does not disappear*. *It leaves a mark as if you were burned by fire and even worse*. *Look at my own hand for example*. *I have a leishmania scar and that is another scar from a burn*. *There is no difference*! *Because of the burn the muscles located under the skin also disappeared*.*”* (FGD22 Men-Allopathic).

Several participants expressed anxiety about CL, especially that it would affect their children. *“We are afraid that this disease would come back in the future because the mosquito is still there*.*”* (FGD21 Women-Folk). In addition, three participants worried about the possible transformation of the scars into skin cancers in the near future. *“…maybe this scar will change in the future into a bad disease [cancer]”* (FGD04 Women-Folk).

Contrastingly, some individuals in the male groups considered CL as a benign problem as it is not contagious and not fatal. One of them said *“An epidemic of leishmania occurred in the past*, *there were many complaints*. *Now people forgot this disease*. *Nowadays if you ask someone something about leishmaniasis*, *he or she will say (s)he does not know anything about it…”* (FGD14 Men-Folk). Moreover, several participants, mainly in Tinghir, raised other health issues which were of more direct concern to them than CL. Access to obstetric care was definitely considered a bigger problem in these remote rural villages than CL.

### Gender discrimination

In these rural societies marriage is considered as a goal in life and an award for every woman. All FGD respondents highlighted that a young woman risks never to get married if her face is affected by CL. *“At work*, *it is not a problem but for the marriage*, *a man will see the scar on the girl’s face and he will go away*. *It’s possible that the girl will never get married”* (FGD01 Men-Allopathic). This was even more clearly expressed by the women in the folk medicine tradition-FGDs who said they would stay polite with a young woman affected by CL and would share household chores such as cleaning and cooking but that this did not imply they agreed to get their sons married to her. *“It’s ok for day-to-day exchanges*, *some polite words*, *good morning*, *good night*. *We pay attention not to hurt her feelings*. *But marriage? No way*.*”* (FGD04 Women-Folk). Others pointed to CL being a reason for revoking marriage vows. “*There was the case of the girls who were engaged and later got this disease*. *They were told*, *we stop [the engagement]*, *go treat yourselves and get cured before we can go on with the marriage*. *These girls were affected either on their faces or on their foreheads*.*”* (FGD20 Women-Folk). As stated above, some male respondents held contrasting views and considered that: *“this was not a huge problem if the girl will never get married”* (FGD01 Men-Allopathic). It seems also that within the context of customary intra-family marriage, the man cannot reject the woman for this reason. Interestingly as well, CL scars that would appear after marriage are no reason for divorce. *“The husband cannot divorce his wife if she is affected by this disease*. *Because she became ill in his house*.*”* (FGD19 Men-Folk).

The extent to which CL leads to social stigma beyond limiting the marriage prospects for women is not uniform. In both provinces, people incriminated the nearest locality to have had the disease earlier before contaminating them via mosquito or rodents. Participants from the area where the disease first appeared in Errachidia mentioned strong social rejection by other groups in the neighborhood. Some said to be ashamed to live in a village with CL *“Men and women of the neighboring locality start insulting us and laughing at us*.*”* (FGD28 Men-Folk). Some feel also rejected by their relatives living abroad who contracted CL during their holidays in Morocco.

Some women affected by CL tend to hide their lesions for fear of stigmatisation. Others felt uncomfortable to share a meal with somebody with an open sore, mostly for fear of contamination. Nonetheless, many participants described a change in attitudes over time, towards less discrimination *“At the beginning*, *many persons who saw me told me I must remove this*. *My face became less beautiful than before*. *I was also upset*. *Every time you touch it*, *you feel something on your face*. *You feel it and it is disturbing*. *After a while you forget its presence”* (FGD04 Women-Folk).

Several times the male respondents expressed they did not see any reason for social rejection, as it is quite common to see scars from various causes in everyday life in this rural context. A scar becomes a part of the person and the society is accustomed to it. Moreover, CL does not change their personal relationships and interactions. *“What is the matter to have an additional scar? It's normal*!… *Even for women*, *it is the signature stamp of our area*.*”* (FGD01 Men-Allopathic).

### Health care seeking patterns

Everybody had an opinion about CL treatment, best summarized as ‘*we try everything*, *but nothing works’*. More than thirty ways of self-treating the skin bumps were cited including food items (honey, oil, eggs, saffron, salt, tea, vinegar, chillies), herbs (henna, Aloe-Vera, fenugreek, basil, rose water), but also harmful products (white spirit, gasoline, tar, bleach water, crushed outdated pills) or alcohol and soap. People try to get rid of the bump by scratching, cutting or burning it. Folk healers often apply burning as well, e.g. using sulfur powder. Besides, they apply water from natural sources, sand and soil to cure the lesion. The ulcers are treated differently, with different mixtures, such as dust, hair and crushed glass, or salt and tobacco. Scars are treated with a long list of different products (lemon to lighten the color, several oils such as oil of cade, but also used motor oil, white spirit, rose water, salt,…).

Conventional treatment in health facilities was a matter for debate. Participants noted that protocols varied, from ointments to a variable number of injections. Some worried about side effects *“They said that the injections have bad effects on the kidneys”* (FGD09 Men-Folk).

Though some acknowledged you have to be treated early to see some effect, several pointed out that the results were often disappointing *“She said to me that she followed the treatment for four weeks every two days per week*. *But now after the end of those treatments she has a blue color*.*”* (FGD02 Women-Allopathic). *“Do you see the spot on the hand of this man sitting near to me*, *even if it appears like it is healed the margins are full of the disease*, *if you touch it*, *it is not yet cured”* (FGD09 Men-Folk). The demand for scar treatment was more pronounced in Errachidia than in Tinghir province.

Mostly, respondents highlighted the lack of effective remedy for the disfiguring scars. *“I tried everything to make the scar disappear*. *I became like an herbalist*. *Until I get tired and I accept the presence of this scar on my face*. *For me cosmetic surgery is dangerous*, *it may even lead to the development of cancer*. *So better leave the scar where it is*.*”* (FGD24 Women-Allopathic). Very few participants, mainly from the allopathic groups, said progressive healing is possible, especially in children who get early medical treatment.

Some respondents emphasize that cosmetic surgery is not accessible and not affordable *“…the specialists in dermatology are far away*. *You should go to another town*. *The doctors there bite you more than the mosquito-bite (Laugh)*. *People are different*, *there are some who have money and some without*. *Then*, *many people accept the reality and ignore the presence of this disease*. *They use natural treatments which is more economical and better than looking for aesthetic surgical interventions”* (FGD08 Women-Folk). Moreover, cosmetic surgery was considered of little benefit compared to the huge cost. *“Leishmania made muscles under the skin disappear*. *That is why there are doctors who say that even cosmetic surgery is not going to give good results for the scar of leishmania”* (FGD22 Men-Allopathic).

## Discussion

Our extensive discussions with members of these rural Moroccan communities learned us that CL is definitely not the main health problem in these remote areas, but nonetheless generates substantial suffering, mainly in women, due to the disfiguring skin outcomes and the social stigma surrounding it. Our respondents described the psychosocial impact of CL as more severe than that of acne and as similar to that of scar tissue from burns. We observed substantial gender differences related to this perceived burden. Women bear the highest burden of CL, as beyond the physical discomfort of active lesions on hands etc., they seem more stigmatized. The emotional well-being of young single women with facial lesions was said to be strongly affected by CL scars, and this corroborates our earlier findings in adolescent youth [19]. Moreover, our respondents considered women at higher risk for CL due to their agricultural and household chores.

The explanation used by these rural community locates the origin of CL clearly in the natural world: the causal link between the insect bite and the skin lesion is made very clearly and specifically, even if the insect is mostly (and erroneously) identified as a mosquito, whereas it is in fact a sand fly. Risk factors for CL are also very correctly identified as all factors that increase vector abundance. One could ask if the link with rodents is made sufficiently strong in the *L*.*major* areas. Unfortunately, there is so far, no effective and sustained community involvement in CL control. Any ongoing reservoir or vector control interventions target perceived immediate risks, like the threat of wild rodents to the agricultural production or the insect nuisance preventing a quiet night sleep in summer days.

At closer examination of the notion of stigma surrounding CL, it seems that this is mostly linked to scars on the face, and then mostly in young women. This type of lesions is only a small subgroup of all the CL lesions. In our study CL seemed a lesser problem in the group of users of “folk medicine”, as they seem to care less about the scars. Due to their very harsh everyday life conditions another scar may then make little difference. The main contrast between users of the folk and allopathic tradition was that the latter considered the CL scars as an unsatisfactory sequela requiring an effective medical solution. It may be interesting to conduct further research on differences in perception according to causative species. The larger size of CL and CL scars may generate more concern in the *L*.*major* than in *L*.*tropica* areas, though the latter lesions are more protracted.

Health seeking behavior patterns for CL and CL scars were multiple and overlapping encompassing the health care traditions of self-help, folk and allopathic medicine. The effect of the toxic and abrasive products and burns used in self-remedies and by traditional healers on the extent of scarring should not be underestimated, and there is a scope for better information and education of the community in this field.

Our findings are consistent with the few available qualitative studies on CL from elsewhere in the world. In Kabul, Afghanistan, women were at higher risk than men to contract CL and affected people were excluded from social life leading to emotional and physical isolation. Similarly to our findings, women faced difficulties to get married if they had a CL lesion or CL scars [24]. Likewise, in Yemen, a review emphasized the stigmatizing potential of disfiguring CL in women [25]. In contrast, in Suriname, where CL mainly affected men, the only factor that generated stigma was the presence of a big (or multiple) CL lesion(s), but no barriers to marriage were evoked [26]. Contrastingly, mucocutaneous CL in Latin America was linked to constant stigma and permanent psychological effects [27–31].

Our qualitative approach derives its strength from a conceptual framework, with an iterative process used in the development of the data collection tools, and the pursuit of saturation in the information reached with the use of additional FGD. The findings from this set of FGD combined with informal interviews and observations on the ground is considered by our team more valid to capture the socio-cultural diversity in interpretations of CL-illness than the use of a restricted number of in-depth interviews with former or actual CL patients. Our study had some limitations though. Some statements by respondents may have been subject to social desirability bias, e.g. when people were informed that the moderator works as a researcher in a national public health institute, this triggered their interest about national health policies and specific health needs in the area. However, we do not think it had a major effect on the way the groups voiced their perception of CL. Secondly, our categorisation of FGD into preferred health care tradition (folk medicine versus allopathic) was not mutually exclusive and may not have captured the full spectrum of care traditions, but it certainly helped us to explore the community perspective across a range of socio-cultural groups in this context.

In conclusion, this qualitative study is the first in Morocco to address the perspective of the population on CL and CL management. As all qualitative research, its main limitations lie in the generalizability or what is called its external validity. Said otherwise, to what extent are our findings representative for other provinces in the country and region? As our findings are consistent though with a larger body of evidence from quantitative KAP studies and psychological assessments by questionnaire surveys and with qualitative research based on FGD conducted earlier in Afghanistan and Surinam, we believe the findings are robust and should inform policy. CL is a true problem in this region, very much intertwined with gender discrimination and lack of access to basic health care in remote rural areas. In dialogue with the community, sound prevention and control policies should be designed. One of the main implications of our findings was that innovation in CL early diagnosis and treatment is desperately needed. The management of existing disfiguring CL scars remains also a subject of further research. In summary, CL is well known but not considered as a major health priority by these poor rural communities in South-eastern Morocco with lots of competing health problems. Its psychosocial impact is substantial though and can be in some cases very important, especially for young single women with facial scars, generating social and self-stigma and diminishing their marriage prospects. A new treatment for CL with better skin outcomes is urgently needed.

## Supporting information

S1 File COREQ checklist. (DOCX)

S2 File Translated FGD topic guide. (DOCX)

S3 File Original participant information sheet. (PDF)
