# Supplementary material for: “The mosquitoes that destroy your face”. Social impact of Cutaneous Leishmaniasis in South-eastern Morocco, A qualitative study
Source: PLoS One. 2017 Dec 20;12(12):e0189906. doi: 10.1371/journal.pone.0189906 (PMC5738074; doi:10.1371/journal.pone.0189906)
Supplement: S1 File — (DOCX) [file pone.0189906.s001.docx]

**Consolidated criteria for reporting qualitative studies (COREQ):**

**32-item checklist**

Developed from:

Tong A, Sainsbury P, Craig J. Consolidated criteria for reporting qualitative research (COREQ): a 32-item checklist for interviews and focus groups. International Journal for Quality in HealthCare.;19(6):349–57. Available from: http://www.ncbi.nlm.nih.gov/pubmed/17872937

| **No. Item** | **Guide questions/description** | **Reported on Page #** |
| --- | --- | --- |
| **Domain 1: Research team and reflexivity** |  | |
| *Personal Characteristics* |  |  |
| 1. Interviewer/facilitator | Which author/s conducted the interview or focus group? | Issam Bennis |
| 2. Credentials | What were the researcher’s credentials? E.g. PhD, MD | **Issam Bennis**  **MD, MPH,**  **PhD candidate**  **Loubna Belaid**  **anthropo sociologist**  **Vincent De Brouwere**  **MD PhD Professor**  **Hind Filali psychosociologist Professor**  **Hamid Sahibi**  **Veterinary doctor PhD Professor**  **Marleen Boelaert**  **MD PhD Professor** |
| 3. Occupation | What was their occupation at the time of the study? | Researcher at the Institute of Tropical Medicine Antwerp and the National school of public Health Rabat |
| 4. Gender | Was the researcher male or female? | Male reported in Methods |
| 5. Experience and training | What experience or training did the researcher have? | Methods -  The researcher  Has a certificated in Qualitative Mixed Methods in international health from the Institute of Tropical Medicine in Antwerp in 2015 |
| *Relationship with participants* |  | |
| 6. Relationship established | Was a relationship established prior to study commencement? | No |
| 7. Participant knowledge of the interviewer | What did the participants know about the researcher? e.g. personal goals, reasons for doing the research | Participant information sheet and Consent Form |
| 8. Interviewer characteristics | What characteristics were reported about the interviewer/facilitator? e.g. Bias, assumptions, reasons and interests in the research topic | Methods |
| **Domain 2: study design** |  | |
| *Theoretical framework* |  |  |
| 9. Methodological orientation and Theory | What methodological orientation was stated to underpin the study? e.g.  grounded theory, discourse analysis, ethnography, phenomenology, content analysis | Methods |
| *Participant selection* |  | |
| 10. Sampling | How were participants selected? e.g. purposive, convenience, consecutive, snowball | Methods |
| 11. Method of approach | How were participants approached? e.g. face-to-face, telephone, mail, email | Methods |
| 12. Sample size | How many participants were in the study? | Methods |
| 13. Non-participation | How many people refused to participate or dropped out? Reasons? | Methods |
| *Setting* |  | |
| 14. Setting of data collection | Where was the data collected? e.g.  home, clinic, workplace | Methods |
| 15. Presence of nonparticipants | Was anyone else present besides the participants and researchers? | Methods |
| 16. Description of sample | What are the important characteristics of the sample? e.g. demographic data, date | Methods |
| *Data collection* |  | |
| 17. Interview guide | Were questions, prompts, guides provided by the authors? Was it pilot tested? | Methods |
| 18. Repeat interviews | Were repeat inter views carried out? If yes, how many? | No |
| 19. Audio/visual recording | Did the research use audio or visual recording to collect the data? | Methods |
| 20. Field notes | Were field notes made during and/or after the interview or focus group? | Methods |
| 21. Duration | What was the duration of the inter views or focus group? | Methods |
| 22. Data saturation | Was data saturation discussed? | Methods |
| 23. Transcripts returned | Were transcripts returned to participants for comment and/or correction? | Methods |
| **Domain 3: analysis and findings** |  | |
| *Data analysis* |  |  |
| 24. Number of data coders | How many data coders coded the data? | Methods |
| 25. Description of the coding tree | Did authors provide a description of the coding tree? | Methods |
| 26. Derivation of themes | Were themes identified in advance or derived from the data? | Methods |
| 27. Software | What software, if applicable, was used to manage the data? | Methods |
| 28. Participant checking | Did participants provide feedback on the findings? | Methods |
| *Reporting* |  | |
| 29. Quotations presented | Were participant quotations presented to illustrate the themes/findings? Was each quotation identified? e.g. participant number | Results |
| 30. Data and findings consistent | Was there consistency between the data presented and the findings? | Discussion |
| 31. Clarity of major themes | Were major themes clearly presented in the findings? | Results |
| 32. Clarity of minor themes | Is there a description of diverse cases or discussion of minor themes? | Results |
