# Supplementary material for: “The mosquitoes that destroy your face”. Social impact of Cutaneous Leishmaniasis in South-eastern Morocco, A qualitative study
Source: PLoS One. 2017 Dec 20;12(12):e0189906. doi: 10.1371/journal.pone.0189906 (PMC5738074; doi:10.1371/journal.pone.0189906)
Supplement: S2 File — (DOCX) [file pone.0189906.s002.docx]

**Supporting information:** Translated FGD topic guide

This Focus Group is a part of a study for preparation of a PhD titled "Control of cutaneous leishmaniasis in Morocco" at the National School of Public Health in Rabat and the Institute of Tropical Medicine in Antwerp.

The main objectives of this study are to identify the knowledge, attitudes, perceptions and practices of the population around cutaneous leishmaniasis.

I request your consent to participate in this discussion group that will help us to understand your experiences related to this disease. The anonymity and freedom of expression is guaranteed.

You can decide at any time to stop the discussion and to withdraw, or else not to participate in a given theme. 60 minutes will be sufficient to complete this entire discussion.

1. **Familiarity with cutaneous leishmaniasis**

The knowledge of the existence of CL in this geographical area

How can we have scars on the skin?

Do you know a disease called leishmaniasis? Do you use other names for this disease?

How long this disease exists in your community? Is this a new disease that appeared recently?

The presence of family antecedents

Did you suffer from this disease before?

Do you know someone who has suffered from this disease in your surroundings?

Is this disease common in this village? Who are the people most affected?

CL Knowledge

How we can recognize this disease? How do you know you are sick with it?

How is this disease transmitted to humans?

On what part of the body do we find lesions? May we have several lesions at once?

What do you think about the origin of these lesions?

How can we avoid this disease? May we develop several times this disease even after healing?

How long can this disease last before healing?

Therapeutic possibilities followed

How can we heal? Is there a cure?

What kind of treatment exists? Where we can find it?

What are the traditional treatments that can be used?

How much does it cost to get treatment?

1. **Perception of this disease**

*The disease severity*

Is this a serious condition? Is it fatal? Are we afraid for our children to have this disease? What is the impact on daily life?

Is it necessary to treat? Would it be better to prevent? How?

*The aesthetic appearance of the lesion or scar*

Did this disease leave scars? How did they appear? On what body parts?

How can we make them disappear?

*The isolation and stigma*

What are the activities that we cannot do when we are affected by this disease?

How we behave in general with those affected by this disease?

(Working together, living together, taking common meal together, walk with, let the children play with the affected person, can cook for your family, possibility of marriage with him or her).

1. **Preventive practices**

What are the preventive measures you take personally to avoid getting sick? (Mosquito bed-nets, insecticides ...)

What measures does the community take to prevent disease in the village? (Waste collection, sanitation ...)

1. **Service use (Question for formal health users)**

Do you know some traditional healers in the area? Have you visited them before?

Even if you have never been to a healer, what do you know about that? Do you know someone who’s going? Why and for what health problems?

1. **Reasons for not using health facilities for care (Question for Traditional users)**

What are reasons for visiting traditional healer rather than health providers?

For what health problem healers are effective? For what disease?

**Thank you**
