# Supplementary material for: “The mosquitoes that destroy your face”. Social impact of Cutaneous Leishmaniasis in South-eastern Morocco, A qualitative study
Source: PLoS One. 2017 Dec 20;12(12):e0189906. doi: 10.1371/journal.pone.0189906 (PMC5738074; doi:10.1371/journal.pone.0189906)
Supplement: S3 File — (PDF) [file pone.0189906.s003.pdf]

بطاقة معلومات للمواطنين المشاركين في مجموعة التركيز

التمثيلات الاجتماعية لداء الليشمانيا الجلدية في المغرب

الباحث الرئيسي: الدكتور عصام بنيس
المنظمة: وزارة الصحة؛ **المنطقة:** إقليم الرشيدية / تنغير
المتعهد: معهد الطب الاستوائي، انتويرب و المدرسة الوطنية للصحة العمومية في الرباط

مرحبا، اسمي عصام بنيس، أنا باحث بالمدرسة الوطنية للصحة العمومية في الرباط، التابعة لوزارة الصحة .
أدعوكم للمشاركة في المائدة المستديرة التي ستتطلب تقريبا مدة ساعة ونصف، مع سبعة أشخاص آخرين للحديث عن مرض الجلد الذي يعرف باسم داء الليشمانيا. لقد أصاب هذا المرض على مدى السنوات الخمس الماضية العديد من الأشخاص في المنطقة.
إن مشاركتك يجب أن تكون طوعية تماما، ولن يتم دفع أي مقابل مادي لأجلها. لك كامل الحرية لرفض المشاركة
لشرح أفضل، ألتمس منك الاستماع إلى المعلومات المتواجدة بهذه المذكرة والتي ستحتفظ بها في حالة ما إذا كنت ترغب في طرح المزيد من الأسئلة أو طلب توضيحات.

غرض ووصف الدراسة

هذه الدراسة تستهدف فهم أحسن لوجهات نظر المواطنين وطريقة تعايشهم مع مرض الليشمانيا الجلدية.
في هذه الدراسة هناك أكثر من 150 مشاركا ومشاركة من عشر جماعات مختلفة بإقليمي الرشيدية وتنغير.
الأسئلة التي سيتم طرحها لها طابع عام ولن يتطلب مجهودا في فهمها.
ألتمس موافقتكم على المشاركة في احدى مجموعات التركيز مع احترام الجنس الذكور على حدة والإناث على حدة. والذين سيتم اختيارهم بنفس الطريقة وفي نفس المكان.
هذه المحادثة ستدوم لمدة تصل الى الساعة وستجرى بالبناية المجاورة للمركز الصحي (   ).

طريقة إجراء الدراسة

إذا كنت موافقا على المشاركة في هذه الدراسة، سوف يطلب منك أن تذهب إلى تلك القاعة في المبنى المجاور. سنبدأ الحصة بعد الظهيرة على الساعة وسيتم تسجيل الحوار بواسطة الكاميرا أو آلة تسجيل الصوت. كل التسجيلات ستستخدم من اجل تذكر الإجابات من طرفي وسيتم مسحها بعد ذلك.
لن يكون هناك أي عرض للشريط المسجل اينما كان.
نضمن لك حرية التعبير وتسجيل كل ما تدلون به من الأجوبة بطريقة مجهولة دون ذكر اسمكم نهائيا. لديك الحق في التوقف عن المشاركة مؤقتا أو بشكل دائم دون إشعار.
لديك أيضا الحق في عدم الإجابة بشكل كلي أو جزئي على نقط النقاش.
لن يتم جمع أي معلومات شخصية عنك. لن يطلب منك لاحقا أي تحليل مخبري أو عينة.

الفوائد

هذه هي واحدة من أولى الدراسات بشأن التصورات والمعارف والمواقف والممارسات من طرف السكان الذين يعيشون في المنطقة المتضررة من داء الليشمانيا الجلدية في المغرب. النتائج المنتظرة ستكون لها فوائد على الصحة العامة للمجتمع.

لجنة الأخلاقيات

تمت الموافقة على إجراء هذه الدراسة من قبل لجنة الأخلاقيات بمعهد الطب الاستوائي في أنتورب. ولجنة الأخلاقيات بالرباط. بالإضافة إلى تراخيص من وزارة الصحة ممثلة في المدرسة الوطنية للصحة العمومية في الرباط، وقسم علم الأوبئة ومحاربة الأمراض في الرباط.

المشاركة الطوعية

يجب أن تكون مشاركتك في هذه الدراسة تطوعية تماما. إنه اختيارك إذا كنت ترغب في المشاركة أم لا.
لديك أيضا الحق في التوقف عن المشاركة في أي وقت، دون حاجة لشرح السبب.

هل لديك أسئلة حول مجموعة التركيز؟

هل أنت على استعداد للمشاركة؟

سوف يتم تسجيل الموافقة الشفهية الخاصة بك في بداية الجلسة.

إذا كانت لديك أسئلة أخرى حول البحث أو في حال كنت تعتقد أنك عانيت من نتائج هذه الدراسة، يمكنك الاتصال الآن، أثناء أو بعد هذا اللقاء بالباحث الرئيسي للدراسة: عصام بنيس
العنوان: المدرسة الوطنية للصحة العمومية، شارع المفضل الشرقاوي، مدينة العرفان. صندوق البريد 6329 الرباط المغرب
الهاتف: +212 537 683 162 الفاكس: +212 537 683 161
